# Food addiction in candidates for bariatric surgery: Association with binge eating disorder and food craving

**DOI:** 10.1192/j.eurpsy.2026.12028

**Published:** 2026-07-31

**Authors:** C. W. Pereira, W. C. N. De Góes Tojal, V. Leao De Melo Neto

**Affiliations:** 1 Faculty of Medicine, Federal University of Alagoas; 2 Arthur Ramos Memorial Hospital, Maceió, Brazil

## Abstract

**Introduction:**

Food addiction (FA) has been increasingly debated as a psychopathological condition associated with obesity. Its overlap with binge eating disorder (BED) raises questions about whether both represent manifestations of the same disorder or constitute distinct clinical entities—and what their main differences might be.

**Objectives:**

This study aimed to investigate the relationship between FA, BED, and food craving in patients with obesity seeking for bariatric surgery, as well as to compare the clinical profiles associated with each condition.

**Methods:**

This study included 50 adults with obesity who were candidates for bariatric surgery. Participants were consecutively recruited between March 2024 and May 2025 at the Obesity Center outpatient clinic of Arthur Ramos Memorial Hospital (Maceió, Brazil). Inclusion criteria were: age ≥18 years, clinical eligibility for bariatric surgery, and signed informed consent. Exclusion criteria were illiteracy or a history of previous bariatric surgery. All patients attending the trial were invited to participate at the time of their medical or psychological consultation. After eligibility screening and informed consent, participants completed a set of instruments in the following order: (1) sociodemographic and clinical questionnaire, (2) modified Yale Food Addiction Scale 2.0 (mYFAS 2.0), (3) Binge Eating Scale (BES), and (4) Modified Trait Food Craving Questionnaire (mFCQ-T).

**Results:**

The mean age was 41.4 years (SD = 11.7), with a predominance of female participants (82%). The average body mass index (BMI) was 39.9 kg/m2 (SD = 5.6), class II or III obesity. The prevalence of food adiction, was 24%. Binge eating, showed a prevalence of 26%. Food craving, evaluated using the mFCQ-T, had a mean score of 12 (SD =9.3). The Kappa agreement between YFAS and BES was 0.307 (95% CI=0.011-0.603). Individuals with FA exhibited a higher prevalence of binge eating (50%) and higher scores on the food craving scale. Additionally, the FA group showed a greater prevalence of regular psychotropic medication use and diagnosed mental disorders. A higher proportion of unemployed individuals was also observed among those with FA. Individuals with BED showed higher scores on both the food craving scale and the food addiction scale, supporting the hypothesis of a partial clinical comorbidity between these conditions, but not a complete overlap. A moderate correlation was observed between food addiction symptoms (mYFAS) and binge eating (BES) (rho =0.577), as well as a strong correlation between FA and food craving (mFCQ-T) (rho =0.726), and between food craving and binge eating (rho = 0.819), all of them with a p value <0.01.

**Conclusions:**

The findings reinforce that, although they share common features, FA and BED are distinct constructs: FA appears to be more related to external triggers (such as ultra-processed foods), while BED is more linked to loss of control overeating.

**Disclosure of Interest:**

None Declared

